# Acceptance of Machine Learning for Medication Selection in Epilepsy to Inform Clinical Trial Design: Co-Design Survey Study

**DOI:** 10.2196/85047

**Published:** 2026-09-25

**Authors:** Madeleine J Smith, Sandra Reeder, Fiona Waugh, Shobi Sivathamboo, Emma C Foster, Daniel Thom, Zhibin Chen, Patrick Kwan, Natasha A Lannin

**Affiliations:** 1 Department of Neuroscience, Monash University, 99 Commercial Road, Melbourne, 3004, Australia, 61 9903 0304; 2 Lived Experience Expert, Melbourne, Australia; 3 Allied Health, Bayside Health, Melbourne, Australia

**Keywords:** machine learning, health care, stakeholder engagement, implementation science, epilepsy, consumer, acceptance, artificial intelligence, AI

## Abstract

**Background:**

Antiseizure medications (ASMs) are the mainstay of epilepsy treatment; however, there is currently no reliable way to predict which medication will be most effective for an individual patient. Machine learning (ML) approaches are increasingly being explored to support personalized ASM selection, but successful implementation will depend on acceptance by both people with epilepsy and treating neurologists. Understanding factors influencing acceptability of ML in clinical decision-making is therefore critical to support engagement, trust, and adherence. The extended unified theory of acceptance and use of technology (UTAUT2) framework has previously been applied to evaluate acceptance of health care technologies, but its suitability for ML-supported ASM selection has not been established.

**Objective:**

The objective of this study was to co-design a UTAUT2-based questionnaire to measure ML technology acceptability that is suitable for use in a clinical trial.

**Methods:**

Adults living with epilepsy and prescribing neurologists were recruited using purposive sampling to participate in a co-design process evaluating the relevance, comprehensiveness, and clarity of the UTAUT2 framework in this clinical context. Participants completed an online survey that collected structured feedback on existing UTAUT2 constructs and identified additional factors influencing ML acceptance. Quantitative responses were analyzed descriptively, and qualitative responses were analyzed thematically to inform adaptation of the framework and development of a context-specific ML acceptability questionnaire.

**Results:**

A total of 32 participants completed the survey, including 22 (68.8%) adults living with epilepsy and 10 (31.2%) neurologists. While participants considered core UTAUT2 constructs relevant, qualitative feedback identified additional domains influencing ML acceptance, including emotional attitudes, perceived risks, knowledge enhancement, conflicts in shared decision-making, and contextual factors such as workplace policy and regulation. On the basis of this feedback, the ML acceptability questionnaire for ASM selection retained relevant UTAUT2 domains and incorporated additional constructs addressing trust and perceived risk. Other themes provided contextual insights for interpretation of ML acceptability and future implementation.

**Conclusions:**

This study highlights the importance of co-design when adapting existing frameworks to the specific clinical context. The co-designed ML acceptance questionnaire for ASM selection developed in this study can be used to evaluate the acceptability of ML technology in the field of epilepsy, strengthening future trials and supporting ongoing technology development.

## Introduction

Worldwide, it is estimated that 5 million people are newly diagnosed with epilepsy every year [1]. The treatment goal following diagnosis of epilepsy is seizure freedom, defined as no seizures for 12 months or more, and is achieved using antiseizure medications (ASMs) [2]. There are over 20 ASMs available, and people with epilepsy may undergo multiple trials of various medications before seizure freedom is achieved [3]. Traditionally, it has been challenging to predict which ASM will be most effective, and often a trial-and-error approach to medication selection can lead to poor seizure control [4], reduced health and quality of life [5], and self-reported fear and anxiety from those with epilepsy as they await effective therapy [6]. The increased availability of digitalized data in health care has led to machine learning (ML) and other AI techniques being used in medicine to improve human clinical decision-making [7,8], including in the field of epilepsy and ASM selection [9,10][11]. Neurologists have applied ML and AI across a range of clinical decisions, from predicting epilepsy risk to monitoring seizures [12]. These applications reflect the broader health care landscape in which health care professionals face increasing demand for diagnostic accuracy, personalized treatment, and automation of administration. ML and AI have been increasingly tested in clinical trials to support clinical decision-making, with most trials in gastroenterology, radiology, cardiology, and other clinical specialties [13]. Similar approaches are now being explored in epilepsy, where prospective clinical trials to determine the clinical efficacy and cost-effectiveness of ML models for ASM selection will provide critical data for personalized medicine. Such a trial is currently underway (ACTRN12623000209695).

In planning clinical trials, the acceptability of ML technology for ASM selection among key stakeholders, including people with epilepsy and treating neurologists, requires particular attention as the acceptability of technology is critical for future clinical implementation [14,15]. Research has shown that the acceptance and implementation of technology often depend on how it is perceived by those using the technology and not on the technology itself [15]. The extended unified theory of acceptance and use of technology (UTAUT2) is a widely used research framework to investigate the acceptability of technology [16] and encompasses the following constructs: performance expectancy, effort expectancy, social influence, facilitating conditions, hedonic motivation, price value, and habit. Importantly, the UTAUT2 is designed to assess anticipated determinants of technology, making it appropriate for evaluating preimplementation acceptance of ML tools in clinical settings. The UTAUT2 has been previously used to measure the acceptance of health care technologies [17-19] and has been applied in AI settings (such as adoption of intelligent elevators [20]). While past studies have shown that the UTAUT2 framework is helpful in understanding user acceptance of health care mobile apps [18,21], prior uses of the framework have focused on acceptance of software or a physical device rather than AI or, more specifically, the use of an ML algorithm for medication selection. Specifically, a gap remains with regard to developing a model specific to ML in epilepsy. Few studies directly address the perspectives of patients and clinicians regarding the use of AI and ML for clinical decision-making.

The overall objective of our study, therefore, was to develop a UTAUT2 questionnaire that is able to capture acceptance and use intentions of ML among adults with epilepsy and prescribing neurologists. This paper presents the co-design process for adapting the UTAUT2 framework and the establishment of clinical utility (comprehensiveness, clarity, and content validity). By including stakeholder perspectives, the findings of this study may inform the design and implementation of ML tools and guide their integration into epilepsy care.

## Methods

### Overview

A participatory research method specific to questionnaire development (as outlined by the South Eastern Sydney Local Health District, [22] was followed. The development of the survey rationale, guided by the interests of those with lived experience, drew from our published work on the perception of ML technologies to support ASM among adults with recent-onset epilepsy [6]. Using these qualitative findings, the draft constructs to assess the acceptability of ML technology for first ASM selection were proposed. Stakeholder members (neurologists and individuals with lived experience of epilepsy) of the project team then recommended developing 2 questionnaires tailored to the experience of the respondent (ie, lived experience participants or neurologist participants). Finally, an online survey method was used to engage with a larger cohort of stakeholders and refine the proposed constructs as well as receive feedback on the content validity of the questionnaire. The survey was distributed via Qualtrics (Qualtrics International Inc) between June 2023 and August 2023, and all respondents provided informed consent.

### Ethical Considerations

Ethics approval was granted by the Monash University Human Research Ethics Committee (project ID 38190). Participants were provided with an online participant information statement outlining the purpose of the study, source of funding, consent to and withdrawal from the study, risks and benefits to participants, confidentiality, storage of data, and dissemination of results. No identifying information was collected, and all responses were anonymous.

### Study Design

The survey was designed in 2 parts: all participants were provided with the adapted UTAUT2 constructs followed by validated feedback questions [23]. As the ML tool had not yet been deployed and the study was conducted prior to clinical trial initiation, participants were asked to consider a hypothetical scenario in which the technology had been used to support ASM selection. Items were primarily phrased in the past tense to simulate postuse evaluation and approximate anticipated experience during trial use; however, present- and future-oriented phrasing was retained for constructs capturing general perceptions and future use. People with epilepsy were presented with the UTAUT2 constructs addressing performance expectancy, facilitating conditions, and behavioral intention. After discussion with the multidisciplinary project team, including members with lived experience of epilepsy, it was deemed that habit, hedonic motivation, social influence, effort expectancy, and price value were not appropriate constructs to include for people with epilepsy. Neurologists were presented with the constructs of performance expectancy, effort expectancy, social influence, facilitating conditions, hedonic motivation, habit, price value, and behavioral intention. For neurologists, price value was assessed using 2 items: a standard 5-point Likert-scale item consistent with the UTAUT2 framework and an additional multiple-choice willingness to pay (WTP) question asking respondents to indicate the percentage of cost they believed would be reasonable to pay. The WTP item was included as a supplementary exploratory measure to contextualize price perceptions. All UTAUT2 constructs were presented along with 5-point Likert scales (“strongly agree” to “strongly disagree”). Given that this study was conducted prior to implementation, constructs were framed prospectively to assess their suitability for integration into a future clinical trial process evaluation. The feedback questions were then presented to assess the comprehensiveness, clarity, and content validity [23] of the UTAUT2 constructs. Feedback questions included whether the constructs were simple and easily understood; whether any items were inappropriate, redundant, or missing; and how likely the questionnaire was to address the acceptability of ML. Participants were presented with questions with Likert-scale (eg, “very unlikely,” “unlikely,” “neutral,” “likely,” and “very likely”) or nominal (eg, “yes,” “no,” “don’t know,” and “unclear”) response formats. Free-text responses were encouraged to gather written feedback on the questionnaire during this development phase.

### Recruitment

People with epilepsy and neurologists were selected as participants representing the 2 key stakeholder groups involved in ASM decision-making. Participants were recruited via email invitations to personal contacts of the project team, advertising from the peak body representing people with epilepsy in Australia (Epilepsy Action Australia, a national community organization for people affected by epilepsy), and social media. Participants were provided with study information, and they self-assessed for eligibility (over 18 years of age, being a permanent resident of Australia, being either a person with epilepsy or a neurologist, and ability to complete the survey over the internet or via telephone call in English).

### Analysis

Multiple-choice items were presented as frequencies and percentages. Feedback from open-ended questions was summarized using inductive content analysis [24]. Discussions were held between 2 researchers (MJS and NAL) to ensure that the analysis provided relevant insights into the content, meaning, patterns, and relationships identified in the free-text responses. Ongoing discussion between the analysts and the broader project team, which included neurologists and individuals living with epilepsy, enabled iterative revision and refinement of the adapted UTAUT2 framework and codevelopment of the questionnaire.

## Results

### Overview

Twenty-two people with epilepsy and 10 neurologists completed the survey. Most respondents (both people with epilepsy and neurologists) found that the draft UTAUT2 questionnaire was directed at the willingness to use ML technology to a moderate or large extent (people with epilepsy: 12/22, 54.5%; neurologists: 6/10, 60%; Multimedia Appendix 1). Participants identified that important issues pertaining to the use of ML technology had been omitted from the UTAUT2 questionnaire, with 45.5% (10/22) of people with epilepsy finding that there were important gaps. Neurologists identified both important gaps (2/10, 20%) and minor gaps (3/10, 30%). Assessing the clarity of the questionnaire, 45.5% (10/22) of people with epilepsy and 50% (5/10) of neurologists reported that the UTAUT2 questionnaire was easy to understand and able to determine the acceptability of ML technology from their perspective.

### Qualitative Feedback on the UTAUT2 Questionnaire

Feedback provided by both people with epilepsy and neurologists on the UTAUT2 questionnaire generated 6 major themes related to design, format, and content.

#### Emotional Attitudes

Participants highlighted that emotional attitudes may impact the acceptability of ML technology for ASM selection, such as apprehension about the use of ML technology. A participant with epilepsy suggested, “Other [emotional] attitude questions may be needed, with Likert response.” Participants suggested that trust was missing and should be included in the questionnaire, with one neurologist stating, “Question of trust in AI technologies?”

#### Risks and Harm

Participants indicated that the questionnaire should include items about the risks of and harm that could be caused by using ML technology for ASM selection, with one neurologist stating that the questionnaire “misses some key aspects of acceptability—specifically around perceived risks and concerns.” Additionally, the potential inappropriateness of the medication selected by the ML technology was raised by participants.

#### Knowledge and Education About ML

ML technology is new and emerging in health care; therefore, both people with epilepsy and neurologists lack prior experience to inform their assessment of acceptability of ML technology. This was reflected in participant responses that stated the importance of providing sufficient information prior to the initiation of the questionnaire. Specifically, participants suggested that they required knowledge about ML technology and its potential in clinical decision-making. A participant with epilepsy stated, “I would like to know more about machine learning” and “What would the percentage accuracy be finding the correct medication?” Additionally, the way clinicians communicate information about ML technology to their patients emerged as a potential influence on acceptability among people with epilepsy.

#### Potential for Conflict Between Clinicians and ML Technology During Decision-Making

Participants recognized the potential for conflict between neurologists and the ML technology in clinical decision-making, with one neurologist stating that there were “no questions on incidents where ML advised a medication they felt was inappropriate for the clinical situation.” A person with epilepsy suggested exploring patient perspectives on collaboration and decision-making dynamics, stating, “Ask patients how they would feel about a doctor collaborating with ML; how they would want the decisions about the medication made i.e., how much sway will be ML versus the doctor and themselves have in the decision.”

#### Context and Future Implementation

Contextual factors influencing the acceptability of ML technology were highlighted by a neurologist, who stated, “Missing questions on what factors would determine acceptability, e.g. patient’s acceptance, employer’s policy, regulatory approval, medicolegal implications.” Additionally, a neurologist suggested that the questionnaire include “questions about balancing cost and utility.”

#### Language and Formatting

People with epilepsy provided varied feedback on the language and formatting of the questionnaire, with one participant indicating, “Questions were straightforward, quick and easy to use.” Conversely, another participant with epilepsy stated, “Some questions were worded in a way I had to re-read a couple of times.” Neurologists noted that more effort should be made to simplify technical jargon, stating that they “recommend plain language and simple sentence structures.”

### Adapted UTAUT2 Framework

The adapted framework (Figure 1) and corresponding survey questions for neurologists (Table 1) and people with epilepsy (Table 2) are shared below.

**Figure 1.**
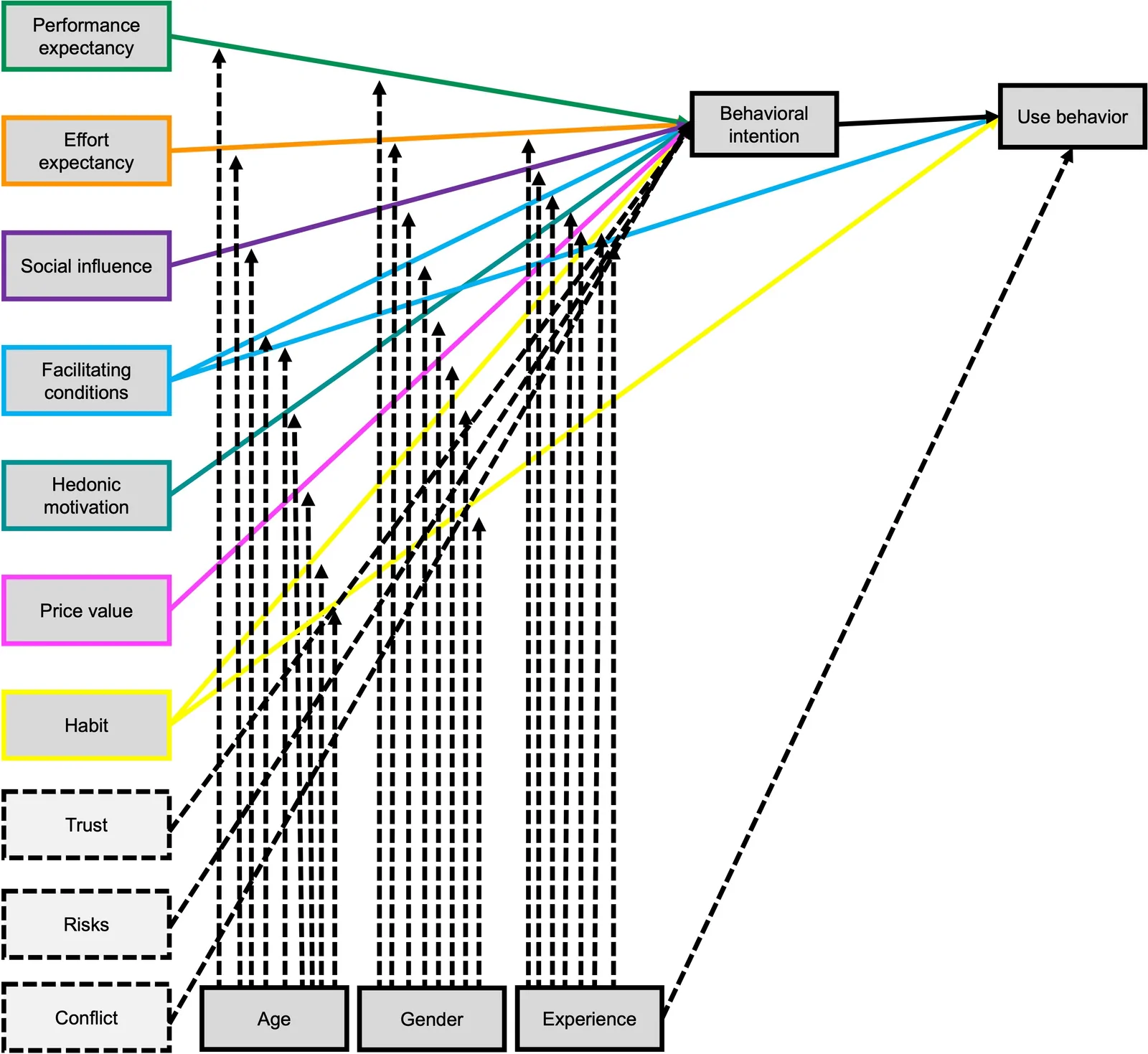
Extended unified theory of acceptance and use of technology (UTAUT2) framework [16] applied to machine learning (ML) in antiseizure medication (ASM) selection for epilepsy. Core UTAUT2 constructs are represented in colored textboxes, whereas additional constructs hypothesized to influence ML acceptance, identified in this pilot study, are shown in dotted textboxes. These constructs will be evaluated in a forthcoming clinical trial of ML in ASM medication selection (ACTRN12623000209695). The moderating effects of age, gender, and experience will also be evaluated.

**Table 1.** Adapted constructs to assess the acceptability of machine learning technology for first antiseizure medication selection among neurologists.

Constructs	Descriptors
Performance expectancy	The first antiseizure medication chosen by the machine learning technology was appropriate for my patient(s). Using the machine learning technology for selecting an antiseizure medication helped to achieve positive clinical outcomes more quickly. The machine learning technology will lead to better outcomes for people with newly diagnosed epilepsy. I agreed with the antiseizure medication selected by the machine learning technology. The machine learning technology prescribed a first antiseizure medication that I would not usually choose.
Effort expectancy	I found the machine learning technology for antiseizure medication selection easy to use. It was easy to explain the machine learning technology to people with newly diagnosed epilepsy. During training, my interaction with the machine learning technology for antiseizure medication selection was clear.
Facilitating conditions	I could get help from others when I had difficulties with any aspect of the machine learning technology. I had the training necessary to integrate the machine learning technology for antiseizure medication selection into my medical practice. I had the resources necessary to integrate the machine learning technology for antiseizure medication selection into my medical practice. The machine learning technology for antiseizure medication selection worked well with other methods I use to select antiseizure medication. I had the knowledge necessary to use the machine learning technology for first antiseizure medication selection.
Social influence	Patients agreed with the use of the machine learning technology for selecting their first antiseizure medication. If the machine learning technology is made available, I would recommend that other neurologists use the machine learning technology for first antiseizure medication selection.
Hedonic motivation	It is exciting and novel to use the machine learning technology for antiseizure medication selection.
Price value	In order to improve antiseizure medication selection, would you be willing to pay an additional price to upgrade your clinic to use machine learning technology? Would you be willing to pay an additional price to use the machine learning technology for antiseizure medication selection if it improved patient outcomes: (1) 1-9% more than your usual methods, (2) 10% more than your usual methods, (3) 11-14% more than your usual methods, (4) 15-24% more than your usual methods, (5) 25-49% more than your usual methods, (6) 50% more than your usual methods, (7) Not willing to pay an additional price to use ML technology.
Habit	The use of the machine learning technology for antiseizure medication selection could become part of my clinical routine.
Trust	I trusted the recommended antiseizure medication generated by the machine learning technology.
Risks	I am concerned about the risks associated with machine learning technology for first antiseizure medication selection. The use of machine learning technology for first antiseizure medication selection could be harmful.
Behavioral intention	I intend to use the machine learning program for antiseizure medication selection if it is made available to me in the future.

**Table 2.** Adapted constructs to assess the acceptability of machine learning for first antiseizure medication selection among people with epilepsy.

Constructs	Descriptors
Performance expectancy	The use of the machine learning technology to select my first antiseizure medication was beneficial for me.
Facilitating conditions	During my appointment with my doctor, the use of the machine learning program was easily incorporated into the discussion about selecting my medication. The information provided to me about the machine learning technology for antiseizure medication selection was clear and understandable. The questions I had about the machine learning technology for antiseizure medication selection were well answered by my doctor.
Behavioral intention	I would accept a machine learning technology used by my doctor that could help select other medication if it was offered to me in the future. I would accept my doctor collaborating with the machine learning technology for my first antiseizure medication selection.
Risks	I am concerned about the risks associated with machine learning technology for antiseizure medication selection.
Trust	I trusted my doctor to make the right decision when selecting my antiseizure medication when using machine learning technology.

## Discussion

### Principal Findings

This study presents an adapted UTAUT2 framework in the context of ML technology for first ASM selection for people with epilepsy. We engaged with key stakeholders (neurologists and people with epilepsy) to collaboratively develop the adapted UTAUT2 framework. This co-design process was conducted in Australia within the context of preparing for a clinical trial, so while our questionnaire is likely to elicit appropriate information regarding the acceptability of ML technology for first ASM selection, modifications and adaptations were made to reflect our clinical context. Consequently, the adapted version includes items about trust, risks, and conflict between clinicians and ML technology in clinical decision-making. These feedback-driven changes to the UTAUT2 model are expected to enhance the assessment of ML acceptability for first ASM selection in epilepsy by capturing key factors influencing stakeholder trust and use intentions. When applied alongside clinical trials evaluating the effectiveness of ML tools, this approach will provide a more comprehensive understanding of both clinical outcomes and end-user perspectives. Together, these insights can inform technology design, guide interpretation of trial findings, and support the safe and effective integration of ML into routine epilepsy care.

### Comparison to Prior Work

The UTAUT2 framework has been extensively used to assess the acceptability of technology [25]. The UTAUT2 questionnaire constructs are performance expectancy, effort expectancy, social influence, facilitating conditions, hedonic motivation, price value, and habit [16]. However, the themes identified in this study extend beyond the original framework and, interestingly, have also been identified in previous studies investigating the acceptability of technology. For example, using the UTAUT2 framework, a study investigating acceptance of mobile marketing found trust and risk to be additional constructs [26]. In a health care context, one study found that trust was a significant predictor of behavioral intention to use a diabetes mobile app among people with diabetes [17]. A review of the factors that facilitate or hinder the implementation of conversational agents in health care found that, similarly to the results of our study, facilitators of implementation extended beyond UTAUT2 constructs to include perceived risk and trust [27]. Additionally, trust is considered a construct in the acceptability of mobile health apps among other patient and clinician groups [17,18,28-31]. Furthermore, participants in our study indicated that whether ML technology or clinician influence was greater in ASM decision-making, could shape their perception of the technology. Existing research on trust in technology automation sheds light on these concerns. In certain circumstances, people may place undue trust in automation even when it is not appropriate, potentially resulting in error or unintended outcomes [32]. These unintended errors or outcomes may erode trust within the community regarding emerging technologies. In the health care context, recent studies have highlighted the specific concerns regarding the integration of AI. A recent systematic review on AI in health care showed that concerns regarding AI include deskilling of health professionals and dependence on technology [33]. Furthermore, a scoping review found that people with cancer generally supported AI but expressed concerns about depersonalization, bias, and data security, with trust influenced by clinician endorsement [34]. In summary, the UTAUT2 framework provides a robust foundation for assessing the acceptability of technologies in health care, although the results of our study highlight the importance of integrating supplementary constructs based on feedback from stakeholders. Specifically, the inclusion of emotional attitudes such as trust may strengthen the comprehensive assessment of ML-supported ASM selection in epilepsy. Although this study was conducted in the context of epilepsy, the adapted framework incorporates broader constructs, including trust and risk, that are relevant across clinical domains. As such, the framework has potential applicability to the evaluation of ML acceptance in diverse health care settings, where similar challenges of integration, transparency, and patient-clinician trust are evident. This enhances the broader value of the adapted UTAUT2 model beyond epilepsy, offering a structured approach to guide the implementation and evaluation of ML technologies in medicine more broadly.

### Limitations

This study has strengths and limitations. Its main strength is the co-design approach that we applied to adapting our proposed model of acceptance and the development of a UTAUT2 questionnaire. However, several limitations should be considered when interpreting the findings. First, this study was limited by a small sample size and convenience sampling methods, both of which may limit the generalizability of the results. Although purposive recruitment enabled inclusion of relevant stakeholder perspectives, the sample may not fully represent broader populations of people with epilepsy and neurologists. Second, the sampling methodology may have introduced selection bias by disproportionately including participants engaged in research. Individuals familiar with research participation may be more receptive to ML-supported clinical decision-making, potentially leading to more favorable assessments of the clarity, relevance, and adequacy of the UTAUT2 questionnaire developed to evaluate ML-supported medication selection. Accordingly, the findings may overestimate the perceived suitability of the questionnaire among broader clinical populations with less exposure to research or digital health initiatives. Future studies should aim to recruit larger and more diverse cohorts with varying levels of research exposure to improve representativeness. Third, neurologists were recruited through existing professional networks, which may have resulted in a relatively homogenous sample with similar clinical perspectives or attitudes. This further introduces the potential for selection bias and may limit the representativeness of the findings across broader neurological practice settings. Expanding recruitment to include neurologists from varied practice settings and geographic regions would strengthen future studies. Fourth, we did not collect demographic information about participants or their prior experience and knowledge of ML technology, which may have impacted their responses. This is particularly important given that age, gender, and experience are established moderators within the UTAUT2 framework. Future studies should collect demographic data to enable subgroup analysis. Fifth, the WTP item should be interpreted cautiously in future applications of this adapted UTAUT2 survey. In public health systems, individual clinicians are rarely responsible for software procurement decisions. Although responses may reflect perceived value, participants may not have direct purchasing authority. Future research should incorporate organizational and health service decision-makers to better evaluate institutional-level adoption and the broader feasibility of ML integration into routine care. Finally, some performance expectancy items use different temporal framing (retrospective, experiential, and prospective) to reflect the different stages of engagement with the ML technology during the planned clinical trial. This design choice may affect internal consistency during psychometric analysis.

### Future Directions

Our context of preparing for a future clinical trial[11] added complexity to this co-design process in that we were preemptively assessing the acceptability of ML technology for ASM selection for epilepsy prior to clinical implementation. Future studies may also seek to include in-depth qualitative interviews with stakeholders in addition to a survey design that limited the nuance of the information collected. Understandably, key stakeholders lack experience and knowledge about emerging ML technologies. The results of this study highlight the importance of engaging with key stakeholders to effectively communicate information about ML technology prior to determining its acceptability. This finding is in line with a recent review that suggests acceptability to be an emergent property of complex knowledge, attitudes, and beliefs, particularly in the context of digital health [35]. In the context of the acceptability of ML technology for ASM, both clinicians and people with epilepsy should be provided with clear, comprehensive, and unbiased information about the effectiveness of the ML technology and any potential limitations prior to the use of the UTAUT2 framework. Health professionals and the general public have indicated that investment in training in and education on AI in health care is necessary [33]. Enhancing the knowledge of all stakeholders involved in the translation of ML technologies into clinical settings will ensure that an acceptable technology is implemented.

### Validation Plan for Forthcoming Trial

As this adapted questionnaire progresses toward deployment in a forthcoming clinical trial, further psychometric evaluation will be important to establish its robustness. In the clinical trial, we will assess internal consistency by construct using the McDonald ω for constructs measured using 3 or more items and the Spearman-Brown coefficient for 2-item constructs (single-item constructs will not be subject to internal consistency reliability testing). Test-retest reliability will be evaluated using intraclass correlation coefficients. Structural validity will be established using exploratory factor analysis across multi-item constructs, followed by confirmatory factor analysis of the overall measurement model. Two-item constructs will be interpreted cautiously and retained in the confirmatory measurement model where model identification permits. Otherwise, they will be analyzed as observed composite scores. Constructs measured using a single item will be treated as observed variables rather than latent factors. Convergent validity will be assessed by examining correlations between the adapted UTAUT2 and an established generalized technology trust scale (Trust in Automation Questionnaire [36]). For interpretation, responses to multi-item constructs on a 5-point Likert scale will be averaged to derive construct scores. Negatively phrased items, specifically those mapping to the risk construct, will be scored in reverse prior to analysis so that higher scores consistently reflect greater acceptability. For price value, the primary scale will be treated as a single-item construct, whereas the exploratory WTP question will be analyzed separately. Finally, the minimal important difference is provisionally set at 0.5 SDs of the baseline score, which will be further validated using anchor-based methods mapped to a global rating of change during the trial.

### Conclusions

This study used a co-design approach to develop a UTAUT2-based questionnaire to assess acceptance of and use intentions regarding ML among people with epilepsy and prescribing neurologists. Developed for use in clinical trials, the questionnaire will provide a comprehensive assessment of influencing factors and will therefore aid in planning for future scale-up of ML technology in the clinical context of first ASM prescription. Ascertaining acceptability is a critical step in the development of new technologies in health care, potentially leading to improved outcomes for people with epilepsy.

## Supplementary material

10.2196/85047 Multimedia Appendix 1 Survey results from the evaluation of the extended unified theory of acceptance and use of technology questionnaire in the context of machine learning for first antiseizure medication selection. People with epilepsy and neurologists rated the questionnaire’s relevance to machine learning use, completeness, clarity, redundancy, and likelihood of capturing factors related to the acceptability of machine learning.

## Abbreviations

ASM: antiseizure medication
ML: machine learning
UTAUT2: extended unified theory of acceptance and use of technology
WTP: willingness to pay
