# Supplementary material for: Acceptance of Machine Learning for Medication Selection in Epilepsy to Inform Clinical Trial Design: Co-Design Survey Study
Source: JMIR AI. 2026 Sep 25;5:e85047. doi: 10.2196/85047 (PMC13618206; doi:10.2196/85047)
Supplement: Supplementary file 1 [file ai-v5-e85047-s001.docx]

**Appendix 1.** Evaluating the UTAUT2 questionnaire in the context of machine learning for first antiseizure medication selection.

| **Question** | **People with epilepsy, n (%)** | **Neurologists, n (%)** |
| --- | --- | --- |
| To what extent are the questions directed at the willingness to use machine learning for first antiseizure medication selection? |  |  |
| Small extent | 3 (14) | 0 (0) |
| Limited extent | 3 (14) | 0 (0) |
| Fair extent | 4 (18) | 4 (40) |
| Moderate extent | 4 (18) | 5 (50) |
| Large extent | 8 (36) | 1 (10) |
| Are there any important issues pertaining to the use of machine learning for first antiseizure selection that should be included which have been omitted? |  |  |
| Crucial gaps | 1 (5) | 0 (0) |
| Important gaps | 10 (46) | 2 (20) |
| Minor gaps | 7 (32) | 3 (30) |
| Minimal gaps | 2 (9) | 2 (20) |
| Insignificant gaps | 2 (9) | 3 (30) |
| To what extent are the questions and response options simple and easily understood? |  |  |
| Small extent | 4 (18) | 0 (0) |
| Limited extent | 3 (14) | 0 (0) |
| Fair extent | 5 (23) | 5 (50) |
| Moderate extent | 6 (27) | 3 (30) |
| Large extent | 4 (18) | 2 (20) |
| How many questions are inappropriate or redundant? |  |  |
| Very many | 3 (14) | 0 (0) |
| Many | 3 (14) | 1 (10) |
| Some | 4 (18) | 4 (40) |
| A few | 6 (27) | 3 (30) |
| Hardly any | 6 (27) | 2 (20) |
| How likely is the questionnaire able to determine the acceptability of machine learning for first antiseizure medication selection? |  |  |
| Very unlikely | 0 (0) | 0 (0) |
| Unlikely | 6 (27) | 1 (10) |
| Likely | 10 (46) | 2 (20) |
| Quite likely | 3 (14) | 6 (60) |
| Very likely | 3 (14) | 1 (10) |
| To what extent are questions likely to elicit information pertaining to the use and experience of machine learning for first antiseizure medication selection? |  |  |
| Small extent | 6 (27) | 0 (0) |
| Limited extent | 2 (9) | 1 (10) |
| Fair extent | 5 (23) | 5 (50) |
| Moderate extent | 4 (18) | 2 (20) |
| Large extent | 5 (23) | 2 (20) |
